# Targeting the Raf kinases in human cancer: the Raf dimer dilemma

**DOI:** 10.1038/bjc.2017.399

**Published:** 2017-12-14

**Authors:** David E Durrant, Deborah K Morrison

**Affiliations:** 1 Laboratory of Cell and Developmental Signalling, National Cancer Institute – Frederick, 1050 Boyles Street, Frederick, MD 21702, USA

**Keywords:** Raf inhibitors, Raf kinases, Ras pathway, ERK cascade, protein dimerisation, cancer therapy

## Abstract

The Raf protein kinases are key intermediates in cellular signal transduction, functioning as direct effectors of the Ras GTPases and as the initiating kinases in the ERK cascade. In human cancer, Raf activity is frequently dysregulated due to mutations in the Raf family member B-Raf or to alterations in upstream Raf regulators, including Ras and receptor tyrosine kinases. First-generation Raf inhibitors, such as vemurafenib and dabrafenib, have yielded dramatic responses in malignant melanomas containing B-Raf mutations; however, their overall usefulness has been limited by both intrinsic and acquired drug resistance. In particular, issues related to the dimerisation of the Raf kinases can impact the efficacy of these compounds and are a primary cause of drug resistance. Here, we will review the importance of Raf dimerisation in cell signalling as well as its effects on Raf inhibitor therapy, and we will present the new strategies that are being pursued to overcome the ‘Raf Dimer Dilemma’.

## Importance of Raf dimerisation in normal and oncogenic signalling

**Figure 1:**
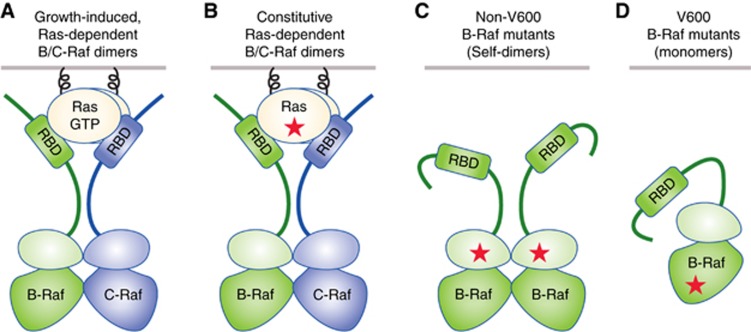
**Raf dimerisation in normal and oncogenic signalling.** (**A**, **B**) Ras-dependent Raf dimerisation is required for Raf kinase activation mediated by normal growth factor-induced signalling (**A**) and by oncogenic signalling driven by mutant RTK or Ras proteins (**B**). In these cases, B-Raf/C-Raf heterodimers predominate. (**C**) Constitutive Ras-independent self-dimerisation is required for oncogenic signalling mediated by non-V600 B-Raf mutants. (**D**) In contrast, V600 B-Raf mutants can signal as Ras-independent monomers. RBD=Ras-binding domain.

The Raf kinases are best known for their role in Ras pathway signalling – a pathway widely utilised to control many cellular processes, including proliferation, differentiation and survival. All members of the Raf family, which include A-Raf, B-Raf and C-Raf, possess a Ras-binding domain (RBD) in their N-terminal regulatory region and can function as effectors of active GTP-bound Ras (Lavoie and Therrien, 2015). The direct interaction with Ras recruits the cytosolic Raf kinases to the plasma membrane and disrupts their autoinhibited state. Ras binding also promotes changes in Raf phosphorylation and induces Raf dimer formation, which is now recognised to be a required step in the Raf activation process. The Raf kinases can dimerise with any of the other Raf family members, and although the factors that determine the dimerisation preferences are poorly understood, B-Raf/C-Raf heterodimers predominate in Ras-dependent signalling (Figure 1a) (Weber *et al*, 2001; Freeman *et al*, 2013b). The Raf kinase domains contact one another through a side-to-side dimer interface, and as a result of protomer transactivation, a catalytically active kinase conformation is assumed, resulting in the phosphorylation and activation of MEK. MEK, in turn, phosphorylates and activates ERK, which plays a critical role in the forward transmission of signals and contributes to inhibitory feedback loops that control the duration and amplitude of pathway signalling. With respect to the inhibitory feedback regulation, active ERK phosphorylates multiple components of the Ras pathway, including the Sos guanine nucleotide exchange factors and members of the Raf family. These phosphorylation events have an overall effect of attenuating signal transmission and, in the case of Raf, disrupt both Ras binding and Raf dimerisation (Dougherty *et al*, 2005).

In human cancer, mutations in receptor tyrosine kinases (RTKs), Ras family members, or the B-Raf kinase frequently hijack the proliferative and survival functions of Ras pathway signalling to drive tumourigenesis. When the pathway is co-opted by constitutive RTK or Ras activity, dimerisation of the wild-type Raf proteins is required to activate MEK and ERK, with B-Raf/C-Raf heterodimers again predominating (Figure 1b). However, when oncogenic signalling is driven by B-Raf, the requirement for Raf dimerisation can vary (Figure 1c and d). Of the Raf kinases, B-Raf has the highest intrinsic kinase activity and is mutated in 50–70% of malignant melanomas, 40% of thyroid carcinomas, 30% of ovarian tumours, and nearly 100% of hairy cell leukaemias (Turski *et al*, 2016). Although many B-Raf alterations have been detected in human cancer, mutations affecting the valine residue at amino acid position 600 (V600) are most prevalent, with V600E-B-Raf accounting for 80–90% of B-Raf mutations in melanoma. V600 mutations allow B-Raf to adopt an active kinase conformation in the absence of dimerisation and, as a result, these mutants can signal as Ras-independent monomers (Haling *et al*, 2014; Yao *et al*, 2015). With limited exceptions, all other oncogenic B-Raf proteins, including non-V600 mutants and B-Raf truncation or fusion proteins, depend on dimerisation for their transforming activity, which can involve Ras-independent self-dimerisation or Ras-dependent heterodimerisation with C-Raf (Yao *et al*, 2015).

## First generation Raf inhibitors: the dimer dilemma

Early efforts targeting the Raf kinases were focused on inhibitors that showed high specificity towards V600E-B-Raf, given its prominent role as a cancer driver. Vemurafenib and dabrafenib were the first of these drugs to gain FDA approval, and they displayed an unprecedented therapeutic effect in melanomas possessing V600E mutations, with over 90% of patients showing some degree of tumour regression and 50–60% achieving partial to complete responses (Lito *et al*, 2013). Somewhat surprisingly, however, these inhibitors had little activity in tumours possessing RTK or Ras mutations, and they had limited clinical benefit in other cancer types with activating B-Raf mutations. Moreover, despite the impressive initial responses in melanomas with V600E-B-Raf mutations, the majority of patients relapsed within a year due to drug resistance. In addition, some patients with responsive tumours developed secondary malignancies during treatment, most frequently squamous cell carcinomas and keratoacanthomas, many of which possessed activating Ras mutations (Lacouture *et al*, 2012; Su *et al*, 2012).

**Figure 2:**
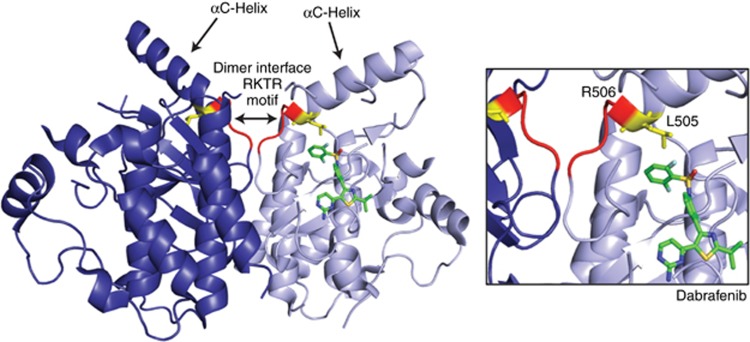
**Structural features of a B-Raf dimer bound to the ATP-competitive Raf inhibitor, dabrafenib.** Shown is a crystal structure of a B-Raf dimer bound to dabrafenib. The αC-helices and the conserved dimer interface RKTR motif are indicated and dabrafenib is depicted in green. The αC-helix regulatory spine residue L505 is shown in yellow and residues comprising the conserved RKTR dimer interface motif are shown in red, with R506 indicated.

Insights regarding these unexpected findings first came from the analysis of melanoma lines expressing either V600E-B-Raf or mutant Ras proteins, where the inhibitors were found to block ERK activation in the V600E-B-Raf lines, but paradoxically activate ERK in Ras mutant cells (Gibney *et al*, 2013). Subsequent studies revealed that drug resistance to vemurafenib or dabrafenib could often be attributed to cellular conditions or mutational alterations where ERK activation was dependent on Raf dimerisation. Through extensive structure/function studies, it is now known that an underlying explanation for these findings resides in how these first-generation inhibitors contact the Raf catalytic domain and impact both dimer formation and dimer activity (Figure 2).

## Insights from the B-Raf kinase domain structure

Like other protein kinase domains, the Raf kinase domain is comprised of an N-terminal lobe (N-lobe) and a C-terminal lobe (C-lobe) that are connected by a flexible hinge region. When Raf becomes activated, spatially conserved, hydrophobic residues spanning both the N- and C-lobes align to form two parallel columns, known as the regulatory and catalytic spines (Taylor *et al*, 2015). Alignment of the spines acts to stabilise the lobes and orient key catalytic residues in a closed conformation that is required for enzyme catalysis. The regulatory spine consists of four residues, of which one is a leucine residue in the N-lobe αC-helix (L505 in B-Raf) and one is the phenylalanine residue of the activation segment DFG motif (D595 in B-Raf). In the inactive state of the Rafs, the regulatory spine is disrupted, and in order for the spine residues to align, both the DFG motif and the αC-helix must move inward to adopt the active ‘IN’ conformation. Adjacent to the conserved leucine spine residue in the αC-helix is an arginine residue (R506 in B-Raf) that plays a critical role in Raf dimer formation and is a component of the conserved RKTR motif at the dimer interface (Baljuls *et al*, 2011). As a result, Raf dimerisation is allosterically linked to Raf activation, in that when Raf dimerises, one protomer can transactivate the other, at least in part, by causing the αC-helix to shift to the ‘IN’ position, thereby aligning the regulatory spine and globally stabilising the active closed kinase conformation.

**Figure 3:**
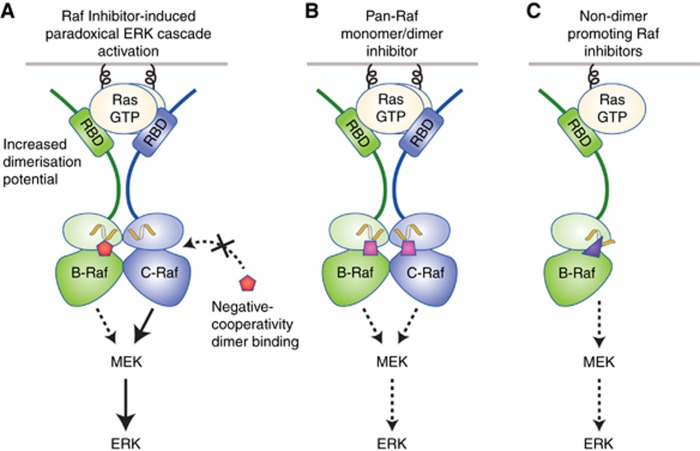
**Effect of Raf dimerisation on Raf inhibitor therapy: overcoming the Raf dimer dilemma.** (**A**) Raf dimerisation can impact the effectiveness of first-generation Raf inhibitors by increasing the dimerisation potential of the Rafs in the presence of activated Ras, thereby promoting paradoxical ERK activation. In addition, all Raf inhibitors in the ‘DFG IN/αC OUT’ class exhibit negative cooperativity in binding to the second protomer of a Raf dimer, making these compounds ineffective inhibitors of dimeric Raf complexes. (**B**, **C**) Efforts to overcome the Raf dimer dilemma have led to the development of compounds that bind monomeric and dimeric Raf with equal potency and inhibit all Raf family members (**B**, pan-Raf monomer/dimer inhibitors) and drugs that alter the position of the αC-helix to such an extent as to prevent/disrupt Raf dimerisation (**C**, non-dimer promoting Raf inhibitors). RBD=Ras-binding domain.

All first-generation Raf inhibitors are ATP-competitive inhibitors, and Raf dimerisation can alter the effectiveness of these drugs through two structurally defined mechanisms. First, all of these inhibitors can increase the dimerisation potential of wild-type Raf proteins in the presence of active Ras (Figure 3a) (Hatzivassiliou *et al*, 2010; Heidorn *et al*, 2010; Poulikakos *et al*, 2010). It should be noted that this phenomenon has also been observed for certain other ATP-competitive inhibitors that exhibit off-target binding to the Rafs, including some inhibitors directed against BCR-ABL, p38, or VEGFR (Lavoie *et al*, 2013). Early studies investigating this effect found that in the presence of active Ras, drug treatment resulted in increased membrane localisation of the Rafs and enhanced Ras binding, as well as increased B-Raf/C-Raf dimer formation (Hatzivassiliou *et al*, 2010; Heidorn *et al*, 2010). Based on these observations, it has been suggested and recently demonstrated that inhibitor engagement destabilises the autoinhibitory interactions occurring between the Raf regulatory and kinase domains, thus promoting the interaction with Ras (Karoulia *et al*, 2016; Jin *et al*, 2017). In addition, given the close proximity of the ATP pocket to the dimer interface, together with the observation that structurally diverse inhibitors can induce purified B-Raf catalytic domains to dimerise *in vitro*, drug binding is thought to restrict the movement of the kinase lobes, thereby generating a static dimer interface that facilitates dimer formation and/or stabilises Raf dimers formed at the membrane (Lavoie *et al*, 2013). More recent studies have revealed that the degree to which these inhibitors promote Ras-dependent Raf dimerisation can be attributed to their differential effects on the αC-helix arginine residue (R506 in B-Raf), which is part of the conserved RKTR dimer interface motif (Karoulia *et al*, 2016).

As with other ATP competitive kinase inhibitors, the Raf inhibitors were initially classified based on whether they bind when the DFG motif is in the active ‘IN’ or inactive ‘OUT’ conformation. Because most of the first-generation compounds were designed to target the active V600E-B-Raf mutant, many are ‘DFG-IN’ binders; however, subsequent structural analysis revealed that some of these drugs, including vemurafenib and dabrafenib, make contacts that stabilise the αC-helix in an inactive ‘OUT’ conformation (Tsai *et al*, 2008; Bollag *et al*, 2010). Of the early Raf inhibitors, vemurafenib and its tool compound PLX4032 were the weakest at promoting Raf dimerisation (Lavoie *et al*, 2013; Freeman *et al*, 2013a), and closer analysis of drug-bound B-Raf structures indicated that in contrast to dabrafenib, vemurafenib displaces the αC-helix to such an extent as to alter the side chain conformation of the R506 residue, thereby disrupting a critical salt-bridge that stabilises the dimeric Raf complexes (Karoulia *et al*, 2016). To date, all Raf inhibitors that do not interfere with the R506 side chain conformation are potent Ras-dependent dimer promoters. Therefore, when inhibitor concentrations are non-saturating, a drug-bound B-Raf promoters can stably interact with Ras and a drug-free Raf promoters, resulting in transactivation of the drug-free Raf promoters and paradoxical activation of ERK. Of note, although the Raf kinases can also form side-to-side dimers with the closely related KSR proteins, dimerisation with KSR does not appear to be a major contributor to the inhibitor-induced paradoxical effect on ERK cascade signalling, in that drug-bound Raf protomers have been shown to have a higher affinity for other Raf family members *vs* the KSR proteins (McKay *et al*, 2011; Lavoie *et al*, 2013). Moreover, the paradoxical activation of ERK induced by the first-generation Raf inhibitors is Ras-dependent (Hatzivassiliou *et al*, 2010; Heidorn *et al*, 2010; Poulikakos *et al*, 2010), whereas the interaction between a drug-bound B-Raf protomer and KSR is Ras-independent and can occur in the cytosol (McKay *et al*, 2011). Thus, depending on the cellular conditions and which Raf inhibitor is used, KSR can in fact act as a competitive inhibitor to reduce the number of drug-bound B-Raf protomers available for binding and transactivating a drug-free Raf protomer at the plasma membrane.

A second way that Raf dimerisation can limit the effectiveness of some first-generation Raf inhibitors is through a mechanism involving negative cooperativity (Figure 3a). More specifically, all compounds that stabilise the Raf kinase domain in a ‘DFG-IN/αC-OUT’ conformation, including both vemurafenib and dabrafenib, are ineffective inhibitors of dimeric Raf complexes (Yao *et al*, 2015; Karoulia *et al*, 2016). In cell-based assays, a much higher dosage of these compounds was needed to inhibit ERK signalling and cell proliferation driven by Ras-dependent or Ras-independent Raf dimers *vs* that needed to block monomeric V600E-B-Raf signalling. Further investigation revealed that when this class of drug binds to one protomer of the dimer, the binding affinity for the second protomer is significantly reduced as is the drug occupancy time. Subsequent structural analysis now indicates that the positioning of the first protomer’s αC-helix in the ‘OUT’ conformation sterically impedes drug binding to the second protomer (Karoulia *et al*, 2016). Thus, this negative cooperativity provides an explanation for why these drugs are ineffective in tumours expressing non-V600-B-Raf mutants that constitutively self-dimerise (Yao *et al*, 2015), and for why the dimeric V600E-B-Raf splice variants often mediate acquired drug resistance in melanomas expressing V600E-B-Raf (Poulikakos *et al*, 2011). Finally, the reduced activity towards dimeric Raf together with the ability to promote Ras-dependent Raf dimerisation provides an explanation for why vemurafenib and dabrafenib have had limited activity in tumour types with upregulated RTK or Ras signalling even when these tumours possess V600-B-Raf mutations (Girotti and Marais, 2013).

## Next-generation Raf Inhibitors: dealing with the dimer dilemma

### Inhibitors of Raf monomers and dimers

The challenge for next-generation Raf inhibitors is to overcome the issues associated with Raf dimerisation and thereby prevent paradoxical ERK activation. One approach that is actively being pursued by many drug discovery programs is the development of compounds that inhibit monomeric and dimeric Raf with equal potency. These drugs typically fall into the ‘DFG-OUT/αC-IN’ binding class, and those that are broadly active against all Raf family members have been termed ‘pan-Raf’ inhibitors (Figure 3b). As expected, all inhibitors in this class promote Raf dimerisation in the presence of active Ras, but due to their ability to bind both protomers of a dimer together with their high affinity for all Raf members, they can inhibit Raf signalling under a broader spectrum of conditions.

Analysis of several pan-Raf inhibitors has been described including LY3009120, MLN2480/TAK-580, CCT196969, CCT241161, and BGB659. In melanoma and colorectal cancer lines, LY3009120 has been found to inhibit MEK activation driven by Ras mutants, V600-B-Raf monomers, or non-V600-B-Raf dimers (Peng *et al*, 2015; Vakana *et al*, 2017). Moreover, this drug can inhibit the activity of B-Raf proteins with in-frame deletions in the β3/αC-helix loop, which are found in ∼5% of pancreatic carcinomas expressing wild-type K-Ras proteins (Chen *et al*, 2016; Foster *et al*, 2016). LY3009120 has also been found to have impressive anti-tumour activity in both cancer line and patient-derived xenograft (PDX) models. Likewise, MLN2480/TAK-580 binds with equal affinity to Raf monomers and dimers and has been reported to inhibit ERK signalling mediated by non-V600 B-Raf dimers (Sun *et al*, 2017). In animal studies, MLN2480/TAK-580 exhibited good brain and tumour penetrance, a property that will be needed to suppress the activity of KIAA1549:B-Raf fusion proteins found in 75% of paediatric low-grade astrocytomas. CCT196969 and CCT241161 distinguish themselves from other pan-Raf inhibitors in that they also possess activity against the Src family kinases (SFK) (Girotti *et al*, 2015). These drugs have been suggested as useful second-round therapies in the treatment of vemurafenib-resistant melanomas, given that many tumours exhibit increased levels of active Src following vemurafenib treatment and that ERK re-activation in drug-resistant cells is often mediated by RTK/SFK signalling.

BGB659 is another drug capable of binding dimeric Raf, but in contrast to the inhibitors described above, BGB659 exhibits selectivity for mutant B-Raf proteins (Yao *et al*, 2015). Under conditions where Ras-GTP levels are low, BGB659 can suppress ERK activation and cell proliferation induced by dimeric B-Raf mutants as well as monomeric V600E-B-Raf, but is less effective in cells with normal to elevated Ras activity, due to its dimer-promoting properties and reduced activity towards wild-type Raf proteins. Nevertheless, drugs like BGB659 may provide a more durable response in melanomas expressing V600E-BRaf, as they would also have activity against the dimeric V600E-B-Raf splice variants that frequently mediate drug resistance to vemurafenib and dabrafenib (Poulikakos *et al*, 2011).

### Non-dimer promoting Raf inhibitors

Another approach to overcome the Raf dimer dilemma includes the identification of compounds that have no ability to promote Raf dimerisation in the presence of activated Ras (Figure 3c). Owing to vemurafenib’s selectivity for V600E-B-Raf and its weaker ability to promote Raf dimerisation, chemists at Plexxikon have used the vemurafenib scaffold to develop novel inhibitors containing substitutions to the drug’s terminal sulfonamide moiety (Zhang *et al*, 2015). A panel of cell lines was then treated with these compounds to identify ones that, unlike vemurafenib, do not promote paradoxical ERK activation in Ras mutant cells, but can still inhibit V600E-B-Raf activity.

This initial screen and subsequent optimisation of compounds led to the discovery of PLX7904, which contains an N-ethylmethyl group in place of the terminal propyl-group of vemurafenib. Structural analysis revealed that the methyl group of the N-ethylmethyl moiety formed closer contacts with the L505 αC-helix spine residue than did the propyl moiety of vemurafenib and stabilised the αC-helix in an extended ‘OUT’ position, which likewise shifted the R506 side chain to a more outward conformation. Moreover, when the N-ethylmethyl moiety was transferred to the dabrafenib scaffold, the resulting hybrid compound induced little paradoxical ERK activation in Ras mutant lines, indicating that this moiety contributes prominently to the paradox-breaking properties of this drug. Given that PLX7904 did not induce B-Raf/C-Raf dimers in melanoma lines with N-Ras mutations, but could inhibit ERK activation in these cells and in melanoma lines expressing the dimeric V600E-B-Raf splice variant, suggests that PLX7904 binding may cause enough steric hindrance at the dimer interface to prevent or disrupt Raf dimerisation.

In preclinical studies, PLX7904 and its optimised analogue PLX8394 had activity in vemurafenib-resistant melanoma lines expressing dimeric V600E-B-Raf splice variants (Zhang *et al*, 2015) and in paediatric astrocytomas expressing dimeric KIAA1549:B-Raf fusion proteins (Sievert *et al*, 2013). Subsequent studies examining the effectiveness of PLX8394 in lung adenocarcinomas, where 40% of B-Raf mutant tumours have non-V600 mutations, found that this drug could inhibit signalling driven by V600 or non-V600 mutants, and could suppress the growth of vemurafenib-resistant lines expressing the dimeric V600E-B-Raf splice variant (Okimoto *et al*, 2016). It should be noted, however, that PLX8394, like vemurafenib, would not be expected to have activity against B-Raf proteins with deletions in the β3/αC-helix loop, given that these deletions restrain the αC-helix in the active ‘IN’ conformation, which would sterically prevent drug binding (Chen *et al*, 2016; Foster *et al*, 2016).

## Alternative approaches to combat the Raf dimer dilemma

While strategies to overcome the Raf dimer dilemma are being incorporated into the design of next-generation Raf inhibitors, exploring alternative approaches to block Raf dimerisation may prove useful. For example, peptides that mimic the B-Raf dimer interface can prevent Ras-driven B-Raf/C-Raf dimer formation and inhibit downstream signalling and cell proliferation (Freeman *et al*, 2013a). Therefore, the development of agents that target the Raf dimer interface could hold promise. Alternatively, because Ras binding is a prerequisite for normal Raf dimerisation and for Raf inhibitor-induced paradoxical ERK activation, the identification of compounds that can disrupt or prevent the Ras/Raf interaction could be highly valuable. Based on previous unsuccessful attempts, many have concluded that the Ras GTPases are undruggable; however, efforts to find Ras-specific inhibitors have been renewed. Towards that end, progress has been made in generating compounds that selectively inhibit the G12C-K-Ras mutant (Lito *et al*, 2016; Ostrem and Shokat, 2016). These drugs form a covalent bond with Cys12 and promote the accumulation of K-Ras in the inactive GDP-bound state, thereby blocking Ras binding to effectors such as Raf, and in turn preventing Raf dimerisation.

Another drug that has been reported to disrupt Ras signalling and Raf dimerisation is rigosertib. Rigosertib was first developed as a non-ATP competitive, multi-kinase inhibitor, and although it exhibits some ability to bind the Raf RBD, this drug is broadly cytotoxic in many cancer lines (Reddy *et al*, 2011). Subsequent examination has revealed that rigosertib as well as microtubule-disrupting drugs such as taxol can engage a stress-induced, phospho-regulatory circuit to shut down Ras signalling (Ritt *et al*, 2016). More specifically, rigosertib activates JNK through mitotic and oxidative stress, and once activated, JNK phosphorylates the RasGEF Sos1 and the Raf kinases on inhibitory sites that, like ERK feedback phosphorylation, suppress RasGTP loading, Ras/Raf binding, and Raf dimerisation.

## Conclusion

Although much progress has been made in understanding the genetic alterations that cause constitutive Ras/ERK cascade signalling in human cancer, identifying therapies that are broadly effective has been difficult. In particular, the usefulness of the first-generation Raf inhibitors has been limited by issues related to Raf dimerisation and paradoxical ERK activation. Next-generation Raf inhibitors take two approaches to address this problem: (1) inhibition of monomeric and dimeric pan-Raf complexes or (2) engagement of the ATP binding cleft in a manner that is inhibitory to Raf dimerisation. While dose limitations and potential side effects of these drugs are yet to be determined, it is expected that like vemurafenib and dabrafenib, the non-dimer-promoting drug PLX8395 will be well tolerated and have little activity in normal cells due to its selectivity for V600E-B-Raf. In contrast, the high potency pan-Raf inhibitors will likely have a reduced therapeutic window due to the fact that they target normal as well as mutant Raf signalling. The pan-Raf inhibitors, in particular, may be good candidates for combinatorial therapies where their activity may synergise with other pathway inhibitors to reduce drug toxicity. Likewise, alternative approaches to inhibit or prevent Raf dimerisation may prove beneficial in the treatment of Ras-driven cancers, or could partner with traditional ATP-competitive Raf inhibitors to augment their efficacy or stave off drug resistance. Thus, with the development of next-generation Raf inhibitors and better strategies to overcome the Raf dimer dilemma, the goal of finding more durable therapies moves forward.
